# Navigating Medical School with AI: A Student’s Perspective

**DOI:** 10.31729/jnma.9072

**Published:** 2025-06-30

**Authors:** Rohit Raj Bhatta, Inesh Khanal

**Affiliations:** 1 Patan Academy of Health Sciences, Lagankhel, Lalitpur, Nepal

**Keywords:** *artificial intelligence*, *education*, *ethics*, *medical student*, *medicine*

## Abstract

Medical school is a strange place where everything feels both deja vu and jamais vu. You've seen this disease before, but somehow, you still have no idea what's going on. Traditionally, we've relied on textbooks, scholarly articles, guidance from senior colleagues, and patient interactions to bridge these gaps but with recent advancements in computational models and algorithms, Artificial Intelligence has been a blessing.

## INTRODUCTION

Medical school is a weird mix of deja vu and jamais vu. You've seen this disease before, but somehow, you still have no idea what's going on. We used to lean on textbooks, scholarly articles, guidance from seniors, colleagues, and patient interactions to bridge these gaps. But, now, with Artificial Intelligence (AI) and its fancy algorithms, we have got a Sherlock to help crack those cases.

Thanks to AI, medical education has transformed in ways we never imagined. However, with everyday use and its widespread adoption, some challenges are coming up, such as ensuring the accuracy of AI-generated information and addressing ethical considerations.^1^

In this article, we will share our own experiences of using AI in medical school, exploring its benefits, challenges, ethical aspects, and the potential it holds for the future of medical education.

## AI AS A STUDY COMPANION

There is no stopping the volume of information in medical school. There's always more. So, studying effectively and using some cheat codes is always helpful. Chatbots serve as an effective tool for interpreting concepts, tables, and graphs, streamlining the learning process._Just prompt the right words, and then you get the answer in a few seconds. AI- powered platforms like ChatGPT, Grok, Anki with AI-enhanced algorithms, and medical question banks have significantly improved the efficiency of our studying which is on par with this study.^2^

Medical textbooks and research papers are rarely short, making it difficult to extract key information quickly. AI tools like Grok, ChatGPT, and other summarization algorithms have helped us condense complex topics into digestible formats while maintaining accuracy. You take a snippet of your Davidson and ask your bot to explain it in let's say two lines and it's ready to go.

And you have those GPTs (Generative Pre-training Transformers) inside the AI chatbots. Personally, after using Ask Robbins - based on Robbins Basic Pathology, Obstetrics, and Gynecology GPT, we realized the potential it holds. Flashcard systems like Anki, enhanced with AI, facilitate spaced repetition - an effective method for retaining vast amounts of information.

## CLINICAL KNOWLEDGE AND DECISION SUPPORT

Beyond academics, AI plays a crucial role in bridging the gap between theoretical knowledge and clinical application. Whether it's learning about disease etiology, diagnosis, or management, AI tools help us gain insights quickly, which is invaluable when you're pressed for time.

Whether to read an X-ray or explain a flowchart, the right prompt in the right chatbot smoothens the wait it takes to give the answers.

We have been using some common prompts for a while now, and they have been quite helpful to us, particularly in our academic endeavors, such as:

1. Can you explain this presentation, slide by slide, with additional information from standard sources?
2. Rephrase my language to sound formal, suitable for an undergraduate medical student, and maybe even add some real-world examples to it.
3. Analyze these two references from PubMed and UpToDate and summarize the key points.

Right prompts and the right resources give you wings. There is a lot of information out there, these AI powered tools simplify it, maintaining the accuracy and pleasing formats.

## CHALLENGES AND ETHICAL CONSIDERATIONS: THE DOUBLE - EDGED SWORD

While AI has greatly enhanced our learning experience, it comes with challenges and ethical concerns:

- Accuracy and Reliability: AI-generated content isn't always accurate, and relying solely on it can lead to misinformation. It's essential to cross-reference AIgenerated content with trusted medical sources. If something feels off, we can simply ask the AI to correct itself. The hallucination of AI is a common problem and while experimenting with writing this article, it used to cite us references out of nowhere.^3^
- Over-Reliance on AI: The convenience of AI makes it tempting to depend on it entirely. We often find ourselves avoiding bulky textbooks in favor of chatbot-driven learning. While it accelerates learning, it's important not to let it break our critical thinking and deep learning.
- Patient Confidentiality and AI Ethics: As AI becomes integrated into clinical practice, ensuring patient data privacy and ethical AI use remains a critical concern. Ethics is what makes us human, otherwise we are just as good as AI chatbots or less. The use of these tools brings challenges, such as the lack of legal protection for individual data. For instance, mental health data collected without consent could later be used in marketing, advertising, and sales by companies.^4^
- Cultural and Geographical Bias: AI systems trained on Western-centric data may not always apply accurately to populations in regions like rural Nepal. AI can show biased data due to its training on specific datasets, making it less reliable in some contexts.^5^

## A DAY IN THE LIFE WITH AI: A NEPALI STUDENT'S ROUTINE

It's 6 AM, and I'm uploading my Acute Appendicitis slides to Grok for a quick summary and extra points. By 9 AM, in class, I'm learning about gloving techniques. Lunchtime brings a skill class on Incision and Drainage, and I'm asking AI for a brief overview. By evening, I'm reviewing Uterine Fibroids - just the essentials. At night, I'm asking AI about my favorite Marvel movie.

AI has become my mentor, my simulator, and sometimes even a friend, always there to make my day easier and my learning smoother.

## THE FUTURE OF AI IN MEDICAL EDUCATION

AI is still evolving, and its role in medical education will only expand. We foresee the integration of AI-driven simulations, virtual patient interactions, and even AI-assisted surgical training.^6^ Personalized AI tutors could revolutionize how medical students prepare for exams and clinical rotations.

The day is not far when we analyze X-rays, MRIs, and pathology slides with high accuracy which is comparable to a medical expert.^7^

While AI enhances learning, it should complement, not replace, traditional education, clinical experience, and human intuition.

## CONCLUSION

Navigating medical school with AI has been a revelation. AI has transformed how we approach medical education from tweaking our study techniques to improving clinical reasoning. However, it is essential to use AI responsibly, ensuring that it remains a tool for enhancement rather than a crutch.

As future doctors, we must embrace AI's potential while upholding the core values of medicine: compassion, critical thinking, and lifelong learning. AI is here to stay, and as medical students, we have the opportunity to harness it for better education and, ultimately, better patient care.
